# Supplementary material for: A T-cell receptor escape channel allows broad T-cell response to CD1b and membrane phospholipids
Source: Nat Commun. 2019 Jan 4;10:56. doi: 10.1038/s41467-018-07898-0 (PMC6320368; doi:10.1038/s41467-018-07898-0)
Supplement: Supplementary file 1 — Supplementary Information [file 41467_2018_7898_MOESM1_ESM.pdf]

Supplementary information

A T cell receptor escape channel allows broad T cell response to CD1b and membrane phospholipids

by Shahine et al.

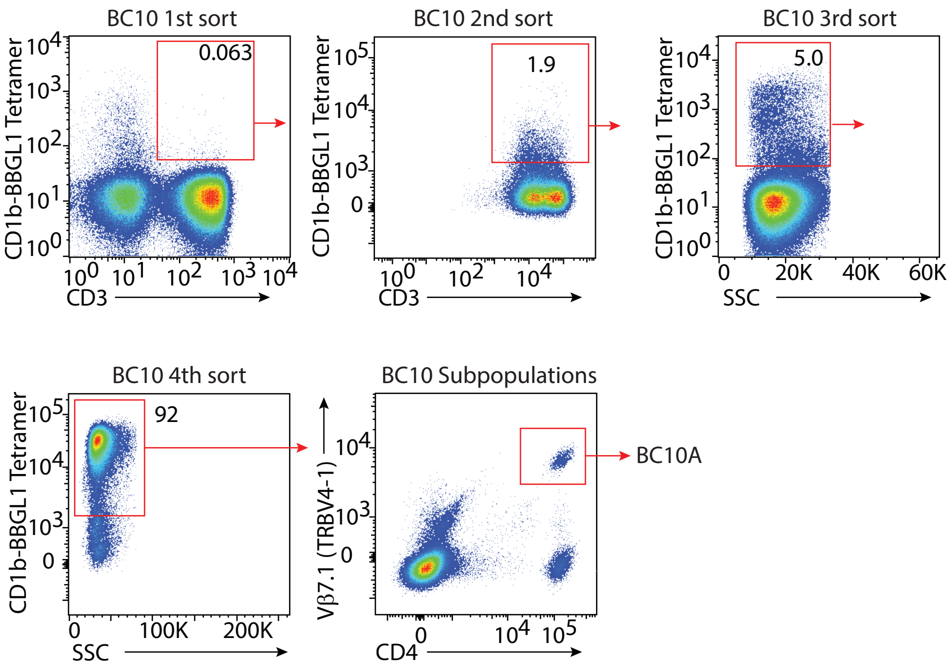

Supplementary Figure 1. Generation of a CD1b-specific cell line from buffy coat 10 (BC10). After four rounds of sorting based on CD1b tetramers treated with BBGL1 lipid, each followed by 14 days of expansion based on stimulation with an antibody against CD3 and irradiated allogeneic feeder cells, cells were gated based on expression of CD4 and TRBV4-1.

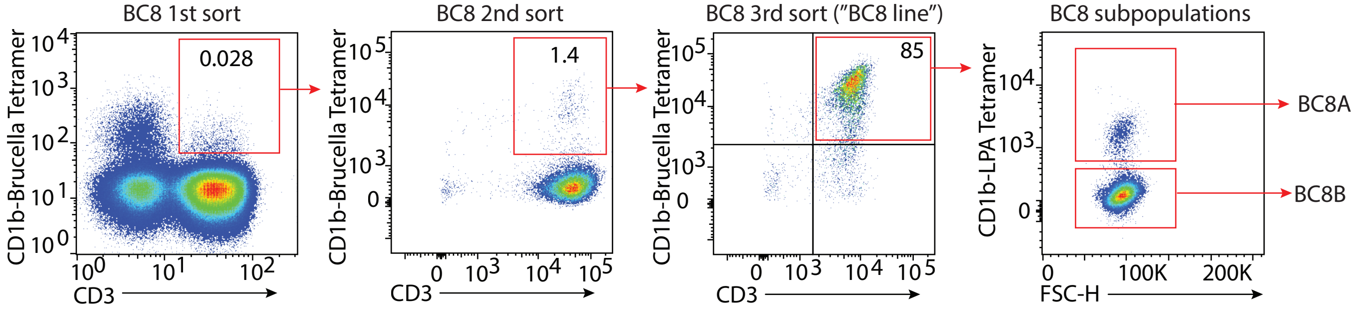

Supplementary Figure 2. Generation of a CD1b-specific cell line from buffy coat 8 (BC8). After two rounds of sorting based on CD1b tetramers treated with Brucella melitensis lipid extract, each followed by 14 days of expansion based on stimulation with an antibody against CD3 and irradiated allogeneic feeder cells, a third round of sorting was based on binding to CD1b tetramers treated with LPA.

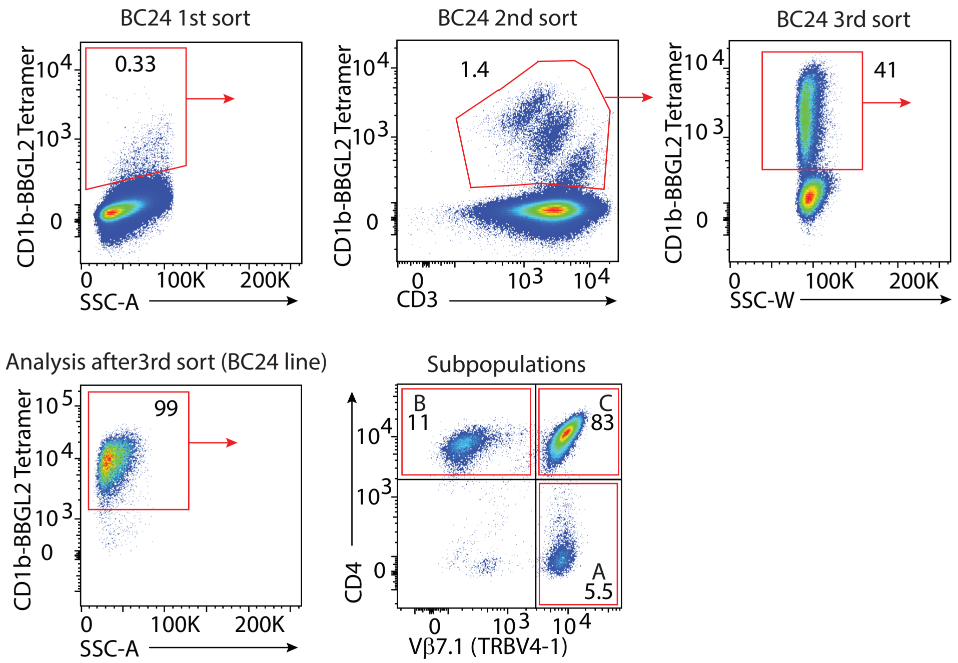

Supplementary Figure 3. Generation of a CD1b-specific cell line from buffy coat 24 (BC24). After three rounds of sorting based on CD1b tetramers treated with synthetic Borrelia burgdorferi glycolipid 2 (BBGL2) lipid, each followed by 14 days of expansion based on stimulation with an antibody against CD3 and irradiated allogeneic feeder cells, subpopulations A, B, and C were gated based on expression of CD4 and TRBV4-1.

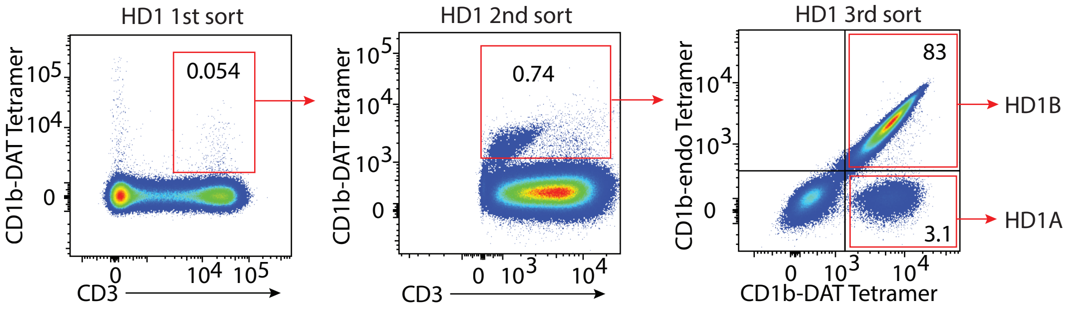

Supplementary Figure 4: Generation of a CD1b-specific cell line from healthy donor 1 (HD1). After two rounds of sorting based on CD1b tetramers treated with synthetic diacyltrehalose (DAT), each followed by 14 days of expansion based on stimulation with an antibody against CD3 and irradiated allogeneic feeder cells, a third round of sorting was based on binding to CD1b-endo tetramers or DAT-treated tetramers. The two subpopulations are named HD1A and HD1B.

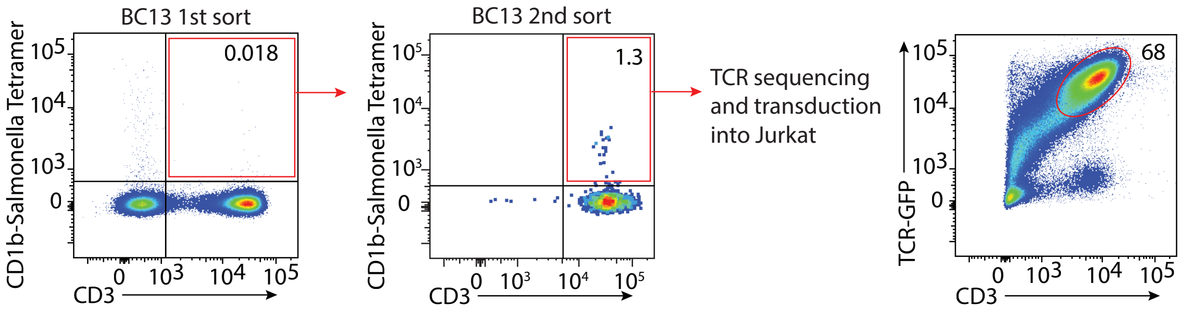

Supplementary Figure 5. Generation of a CD1b-specific cell line from buffy coat 13 (BC13). After two rounds of sorting based on CD1b tetramers treated with Salmonella typhimurium lipid extract, each followed by 14 days of expansion based on stimulation with an antibody against CD3 and irradiated allogeneic feeder cells, a third round of sorting was performed to obtain single cell TCR sequences. The obtained TCR was cloned into a viral construct that also encodes GFP and used to transduce Jurkat76 cells.

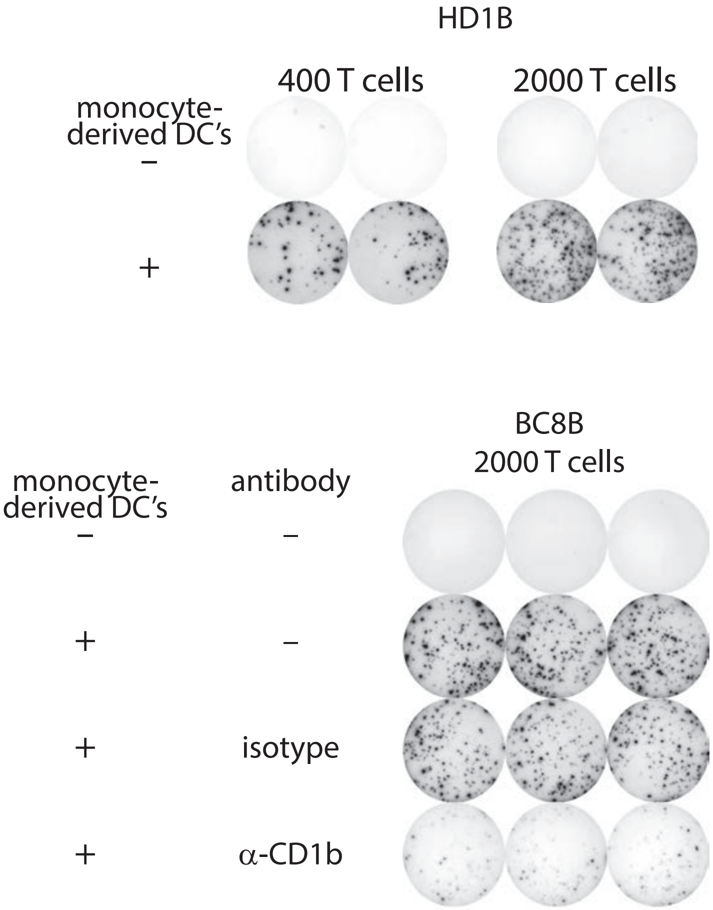

Supplementary Figure 6. Monocyte-derived dendritic cells stimulate CD1b-specific T cell lines. IFN-γ ELISPOT assay of T cell lines HD1B and BC8B cells stimulated with human monocyte-derived dendritic cells that were prepared from primary monocytes. The ELISPOT was performed without the addition of exogenous antigen.

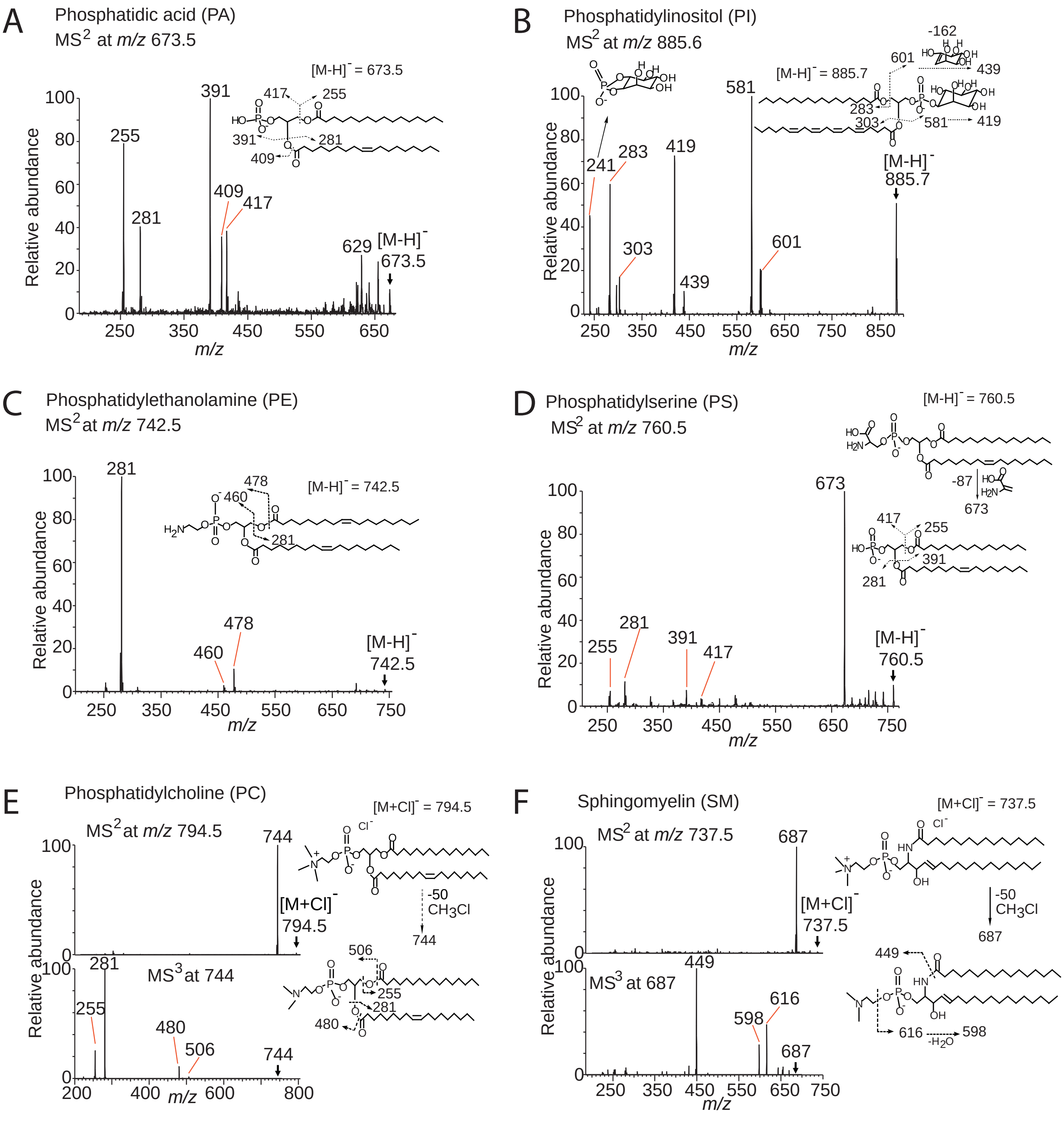

Supplementary Figure 7. Low resolution mass spectrometry of CD1b-endo-bound lipids. Identification of self-lipids eluted from CD1b-endo by negative mode nanoelectrospray ionization collision-induced dissociation mass spectrometry (CID-MS). This figure supports the assignments in Fig. 2a.

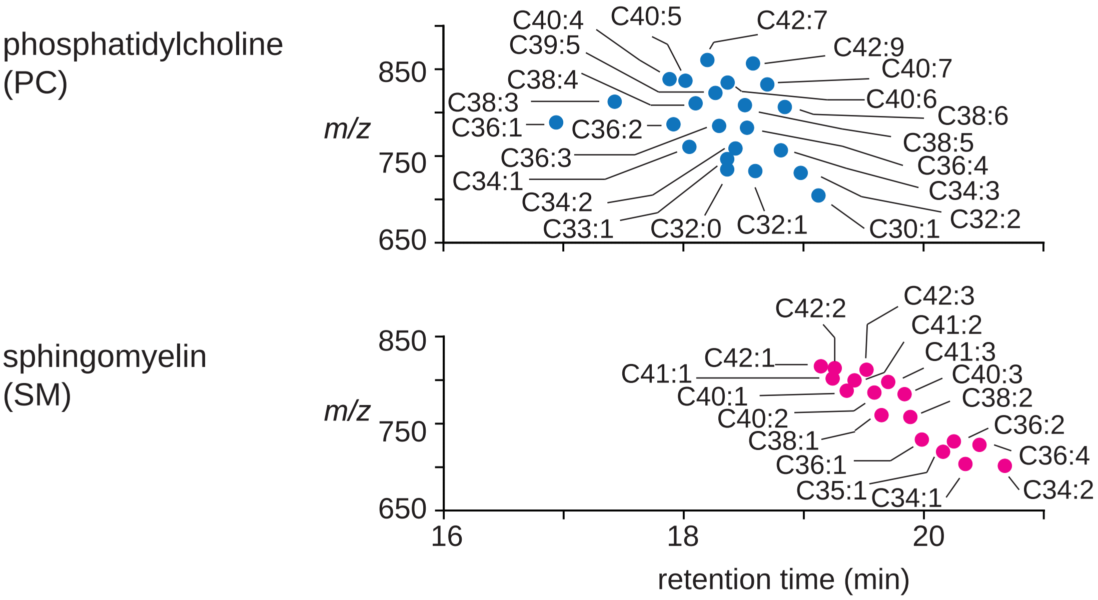

Supplementary Figure 8. High resolution mass spectrometry of CD1b-endo-bound lipids. CD1b eluents were analyzed by quadrupole time of flight (Q-Tof) HPLC-MS. The lipid identities of phosphatidylcholine (PC) and sphingomyelin (SM), were determined based on the accurate mass and the retention time matching those of the lipid standards. Chain length and saturation variants within the same lipid class typically have similar retention times and m/z values and thus appear as clusters. Delineation of PC (blue) and SM (pink) as 22 and 17 molecular species, respectively, presented as 'CX:Y', where 'X' is the total number of carbon (C) atoms in the combined fatty acyl and sphingosine units and 'Y' is the total number of unsaturations.

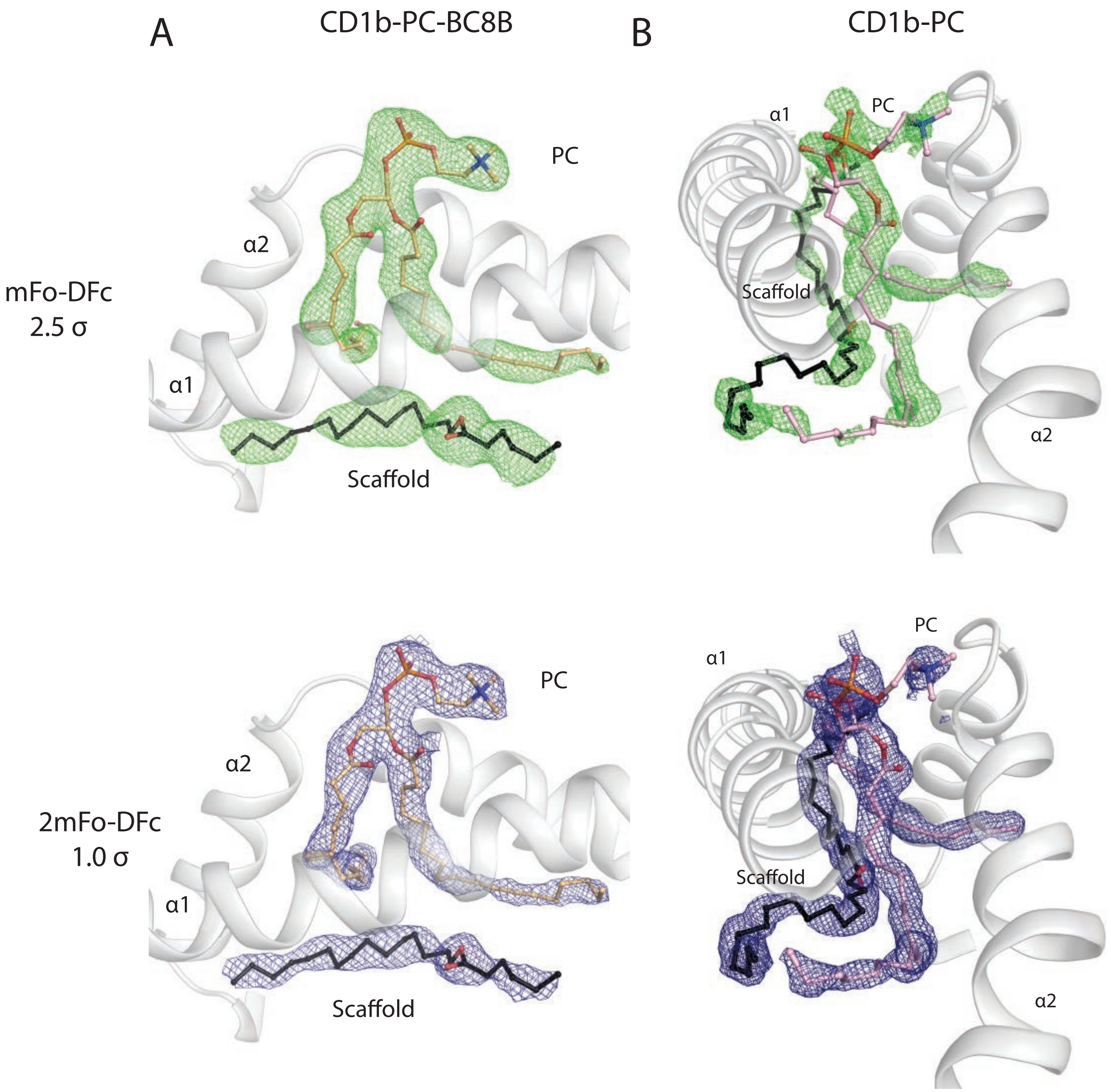

Supplementary Figure 9. Lipid electron density maps. Lipids bound within the antigen binding cleft of CD1b as observed in the crystal structure of (A) the CD1b-PC-BC8B complex, and (B) the CD1b-PC complex. Lipids presented as stick representation, with PC colored A) light orange, and B) pink, with the scaffold lipid colored black. Oxygen, nitrogen and phosphorus are coloured red, blue and orange respectively. Electron density maps are contoured around the lipids within the antigen binding grooves. Omit density maps (mFo-DFc, upper) are coloured in green and contoured to 2.5 σ, and refined density maps (2mFo-Fc, lower) are colored in blue and contoured to 1.0 σ.

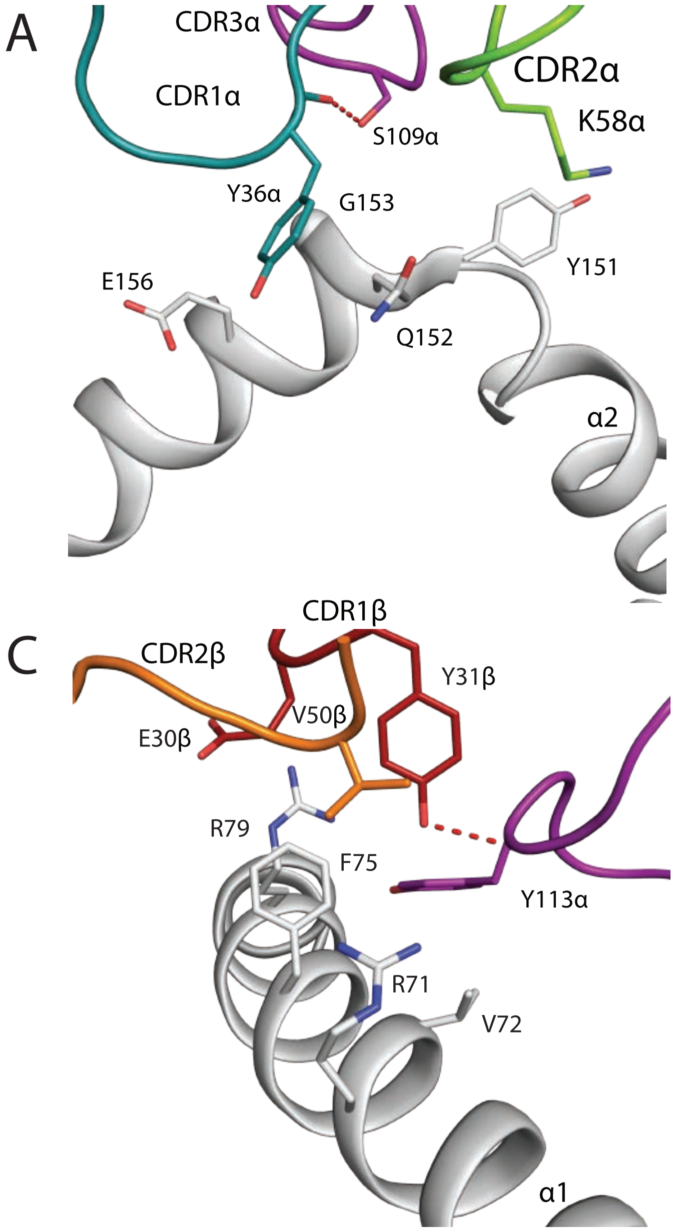

Supplementary Figure 10. Contacts between CD1b and the BC8B TCR. Amino acid contacts between CD1b (grey) and the BC8B TCR A) CDR1α (teal) and CDR2α (green), B) CDR3α (purple), C) CDR1β (red) and CDR2β (orange), and D) CDR3β (yellow) regions. CD1b and TCR backbones are represented as ribbons, with amino acid residues involved in contacts represented as sticks, with the exception of glycine residues, which are represented as spheres. Oxygen, nitrogen, and sulfur are represented in red, blue, and brown respectively. Hydrogen bonds, Salt Bridges, and hydrophobic interactions are represented as red, green, and black dashes respectively.

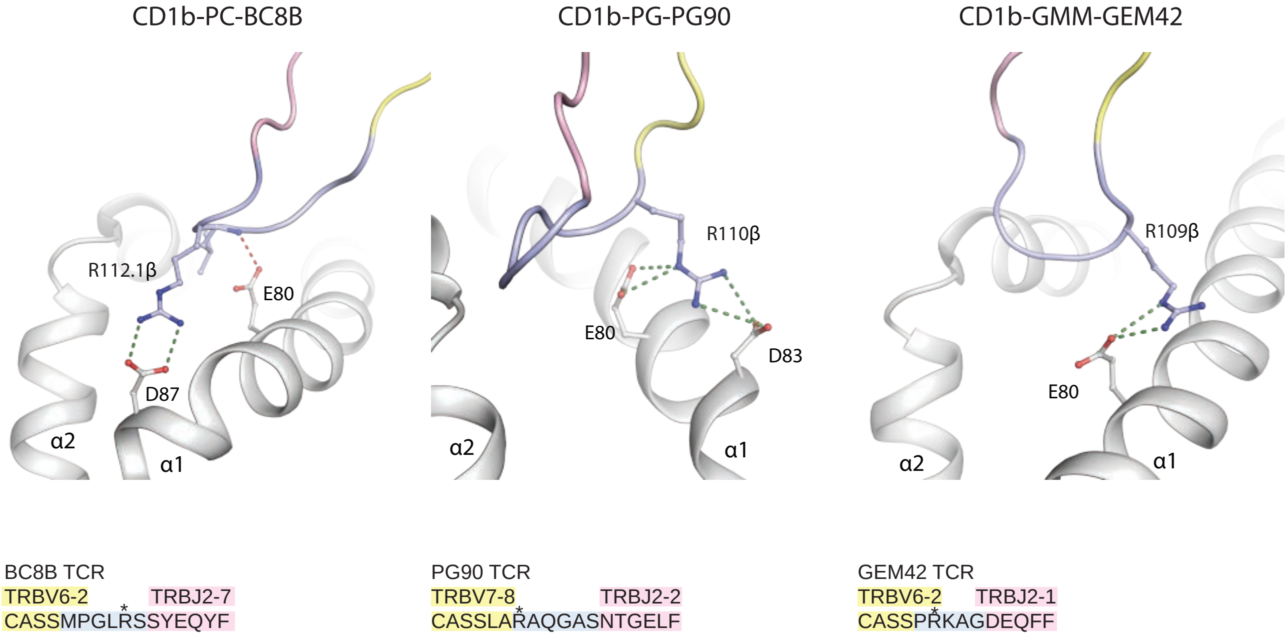

Supplementary Figure 11. CDR3β-CD1b salt bridge contact comparison between the BC8B, PG90, and GEM42 TCRs. The CDR3β regions encoded by variable (TRBV) (yellow), N addition (blue), and junction (TRBJ) (pink) regions, and CD1b (grey), are represented as ribbons. Amino acid residues involved in contacts are represented as sticks. Salt bridges and hydrogen bonds are indicated as green and red dashes respectively. (Lower) TCRβ gene usages for BC8B (TRBV6-2/TRBV2-7, left), PG90 (TRBV7-8/TRBV2-2, middle), and GEM42 (TRBV6-2/TRBV2-1, right) TCRs. Amino acid sequences encoded in the CDR3 regions are indicated, and color coded as above. N-region encoded arginine is indicated by asterix (*).

Supplementary Table 1: Data Collection and Refinement Statistics

	**CD1b-PC-BC8B**	**CD1b-PC**	**BC8B TCR**
**Space Group**	P 1 21 1	P 21 21 21	P 21 21 21
**Resolution Range (Å)**	65.70 – 2.40 (2.53 – 2.40)	46.95 – 1.70 (1.73 – 1.70)	47.29 – 2.02 (2.07 – 2.02)
**Cell Dimensions (Å, °)**	a=73.30, b=65.70, c=101.90<br>α = γ = 90.00, β=100.70	a=57.98, b=80.03, c=92.77<br>α = β= γ = 90.00	a=49.62, b=76.88, c=119.95<br>α = β= γ = 90.00
**Total No. of reflections**	538019 (78785)	707553 (36798)	210004 (15953)
**No. of unique reflections**	37515 (5464)	48143 (4719)	30920 (2266)
**Multiplicity**	14.3 (14.4)	14.7 (14.8)	6.8 (7.0)
**Completeness (%)**	100.0 (100.0)	99.9 (99.8)	100.0 (100.0)
**CC (1/2)**	0.903 (0.500)	0.999 (0.675)	0.995 (0.802)
**R_{pim} (%)** [a]	17.8 (46.5)	4.4 (62.9)	6.0 (60.1)
**Mean I/ σ(I)** [b]	5.2 (2.0)	17.7 (1.9)	9.1 (2.0)
**R_{factor}/R_{free} (%)** [c]	20.9/21.9	17.9/20.9	21.0/22.8
**Non-hydrogen atoms**	6820	3933	3497
**Macromolecules**	6303	3094	3294
**Ligands**	103	301	16
**Water**	418	539	187
**Protein Residues**	803	382	427
**R.M.S.D. from ideality**			
**Bond Length (Å)**	0.007	0.008	0.009
**Bond Angles (°)**	1.050	1.053	1.100
**Ramachandran Plot**			
**Favoured Region (%)**	97.97	99.74	97.10
**Allowed Region (%)**	1.90	0.26	2.90
**Outliers (%)**	0.13	0.00	0.00
**B-Factors (Å^2)**			
**Average B-factors**	31.96	27.92	53.22
**Average Macromolecule**	31.95	24.87	53.12
**Average Ligand**	35.75	44.27	72.40
**Average Water**	31.25	36.31	53.29
**PDB Accession Code**	6CUG	6D64	6CUH

a$R_{pim} = \Sigma_{hkl} [1/(N - 1)]^{1/2} \Sigma_i | I_{hkl,i} - \langle I_{hkl}\rangle |/\Sigma_{hkl} \langle I_{hkl}\rangle$, **b**σ($I$) is the estimated standard deviation of the integrated intensity (I). **c** $R_{factor} = \Sigma_{hkl}||F_o| - |F_c||/\Sigma_{hkl}|F_o|$ for all data except 5%, which were used for R_{free} calculation. Highest resolution shell is shown in parenthesis.

Supplementary Table 2: Contacts between the BC8B TCR and CD1b-PC

**TCR Gene**	**TCR Residue**	**CD1b**	**Bond Type**
CDR1α	Tyr36	Gln152, Gly153, Glu156,	VDW
CDR2α	Lys58-Cβ	Gln152-Nϵ2	VDW
CDR3α	Ser109-Oγ	G153-Cα	VDW
CDR3α	Tyr113-OH	Arg79-NH1, Glu80-Oϵ2	HB
CDR3α	Tyr113	Val72, Phe75, Gly76, Arg79, Glu80	VDW
CDR3α	Gln114-Nϵ2	Val72	VDW
CDR3α	Gln114	Glu68, Val72	VDW
CDR1β	Glu37-Oϵ2	Phe75, Arg79	VDW
CDR1β	Tyr38-OH	Phe75	VDW
CDR2β	Val57-Cγ2	Arg71-Cδ	VDW
CDR3β	Met108-Sδ	Arg79-NH1	HB
CDR3β	Pro109-O	Arg79-NH2	HB
CDR3β	Pro109	Arg79	VDW
CDR3β	Gly110	Arg79, Glu80	VDW
CDR3β	Leu111-N	Glu80-Oϵ2	HB
CDR3β	Leu111	Glu80, Tyr151, Ile154	VDW
CDR3β	Arg112.1-NH1-NH2	Asp87-Oδ1-Oδ2, Asp83-Oδ2-O	SB, HB
CDR3β	Arg112.1	Asp83, Phe84, Asp87, Tyr151	VDW
**TCR Gene**	**TCR Residue**	**PC**	**Bond Type**
CDR1α	Thr28-Cβ	C6	VDW
CDR1α	Gly29	O2P	VDW
CDR1α	Tyr36-N-O	O2P, O1P	HB
CDR1α	Tyr36	O1P	VDW
CDR1α	Pro37-N	O1P	HB
CDR3α	Pro108-C-Cβ-Cγ	C1, C4-C7, C7, O1P, O3P, P	VDW
CDR3α	Ser109-N-O	O1P, O2-O31	HB
CDR3α	Tyr113-Cδ2-Cϵ2	C32, C34	VDW
CDR3β	Leu111-Cδ2	C32, O31	VDW

VDW: Van der Waals interaction (cut-off of 4.0 Å), HB: Hydrogen Bond (Cut-off of 3.5 Å), SB: Salt Bridge (cut-off of 3.5 Å)

Corresponding Author Name: Ildiko Van Rhijn

Manuscript Number: NCOMMS-18-17068

Reporting Checklist For Nature Communications Life Sciences Articles

This checklist is used to ensure good reporting standards and to improve the reproducibility of published results. For more information, please read Reporting Life Sciences Research.

Figure legends

☑ Check here to confirm that the following information is available in all relevant figure legends (or Methods section if too long):

- the **exact sample size (*n*)** for each experimental group/condition, given as a number, not a range;
- a **description of the sample collection** allowing the reader to understand whether the samples represent **technical or biological replicates** (including how many animals, litters, culture, etc.);
- a **statement of how many times the experiment shown was replicated in the laboratory**;
- **definitions of statistical methods and measures**: (For small sample sizes (n<5) descriptive statistics are not appropriate, instead plot individual data points)
  - o very common tests, such as *t*-test, simple χ^2 tests, Wilcoxon and Mann-Whitney tests, can be unambiguously identified by name only, but more complex techniques should be described in the methods section;
  - o are tests one-sided or two-sided?
  - o are there adjustments for multiple comparisons?
  - o **statistical test results**, e.g., ***P* values**;
  - o definition of '**center values**' as **median** or **mean**;
  - o definition of **error bars** as **s.d.** or **s.e.m.** or **c.i.**

This checklist will not be published. Please ensure that the answers to the following questions are reported in the manuscript itself. We encourage you to include a specific subsection in the Methods section for statistics, reagents and animal models. Below, provide the page number or section and paragraph number (e.g. "Page 5" or "Methods, 'reagents' subsection, paragraph 2").

Statistics and general methods

Statistics and general methods	Reported in section/paragraph or page #:
1. How was the sample size chosen to ensure adequate power to detect a pre-specified effect size? (Give section/paragraph or page #)	None of our experiments was designed to draw conclusions regarding a population based on samples of that population
For animal studies, include a statement about sample size estimate even if no statistical methods were used.	NA
2. Describe inclusion/exclusion criteria if samples or animals were excluded from the analysis. Were the criteria pre-established? (Give section/paragraph or page #)	NA
3. If a method of randomization was used to determine how samples/animals were allocated to experimental groups and processed, describe it. (Give section/paragraph or page #)	NA
For animal studies, include a statement about randomization even if no randomization was used.	NA
4. If the investigator was blinded to the group allocation during the experiment and/or when assessing the outcome, state the extent of blinding. (Give section/paragraph or page #)	NA
For animal studies, include a statement about blinding even if no blinding was done.	NA
5. For every figure, are statistical tests justified as appropriate?	Yes
Do the data meet the assumptions of the tests (e.g., normal distribution)?	NA
Is there an estimate of variation within each group of data?	NA
Is the variance similar between the groups that are being statistically compared? (Give section/paragraph or page #)	NA

(Continues on following page)

Reagents

Reported in section/paragraph or page #:

6. To show that antibodies were profiled for use in the system under study (assay and species), provide a citation, catalog number and/or clone number, supplementary information or reference to an antibody validation profile (e.g., Antibodypedia, 1DegreeBio).

   CD4-APC: BD 555349 clone RPA-T4
   CD4-BV421: Biolegend 300532 clone RPA-T4
   TRBV4-1-Fitc: Beckman Coulter IM2408 Vb7.1 clone ZOE
   CD3-Fitc: BD 349201 clone SK7

7. Cell line identity:

   a. Are any cell lines used in this paper listed in the database of commonly misidentified cell lines maintained by ICLAC (also available in NCBI Biosample)?

   NO

   b. If yes, include in the Methods section a scientific justification of their use – indicate here on which page (or section and paragraph) the justification can be found.

   NA

   c. For each cell line, include in the Methods section a statement that specifies:

   - the source of the cell lines

   K562 and C1R cells were stably transfected with CD1 isoforms in house

   - have the cell lines been authenticated? If so, by which method?

   Yes, we analyzed the expression of CD1 isoforms on K562 and C1R cells by flow cytometry

   - have the cell lines been tested for mycoplasma contamination?

   In this checklist, indicate on which page (or section and paragraph) the information can be found.

   No

Animal Models

Reported in section/paragraph or page #:

8. Report species, strain, sex and age of animals

   NA

9. For experiments involving live vertebrates, include a statement of compliance with ethical regulations and identify the committee(s) approving the experiments.

   NA

10. We recommend consulting the ARRIVE guidelines (PLoS Biol. 8(6), e1000412,2010) to ensure that other relevant aspects of animal studies are adequately reported.

Human subjects

Reported in section/paragraph or page #:

11. Identify the committee(s) approving the study protocol.

    Partners Healthcare IRB

12. Include a statement confirming that informed consent was obtained from all subjects.

    We used de-identified discarded white blood cells from blood donations, distributed by the BWH biospecimen bank

13. For publication of patient photos, include a statement confirming that consent to publish was obtained.

    NA

14. Report the clinical trial registration number (at ClinicalTrials.gov or equivalent).

    NA

15. For phase II and III randomized controlled trials, please refer to the CONSORT statement and submit the CONSORT checklist with your submission.

    NA

16. For tumor marker prognostic studies, we recommend that you follow the REMARK reporting guidelines.

    NA

(Continues on following page)

Data Availability

Reported in section/paragraph or page #

17. Provide a Data availability statement in the Methods section under "**Data availability**", which should include, where applicable:

- Accession codes for deposited data
- Other unique identifiers (such as DOIs and hyperlinks for any other datasets)
- At a minimum, a statement confirming that all relevant data are available from the authors
- Formal citations of datasets that are assigned DOIs
- A statement regarding data available with restrictions

See our data availability and data citations policy page for more information.

Data deposition in a public repository is mandatory for:

a. Protein, DNA and RNA sequences
b. Macromolecular structures
c. Crystallographic data for small molecules
d. Microarray data

> Data availability
> Structural data were deposited in the Protein Data Bank, with the following accession codes: BC8B TCR (6CUH) [www.ncbi.nlm.nih.gov/Structure/pdb/6CUH], CD1b-PC-BC8B (6CUG) [www.ncbi.nlm.nih.gov/Structure/pdb/6CUG], and CD1b-PC (6D64) www.ncbi.nlm.nih.gov/Structure/pdb/6D64]. All remaining data are available within the article and its supplementary information files and from the corresponding authors on request. A reporting summary for this article is available as a Supplementary Information file.

Deposition is strongly recommended for many other datasets for which structured public repositories exist; more details on our data policy are available here. We encourage the provision of other source data in supplementary information or in unstructured repositories such as Figshare and Dryad. We encourage publication of Data Descriptors (see Scientific Data) to maximize data reuse

18. If computer code was used to generate results that are central to the paper's conclusions, include a statement in the Methods section under "**Code availability**" to indicate whether and how the code can be accessed. Include version information as necessary and any restrictions on availability.

> NA
